# An Intelligent Data Uploading Selection Mechanism for Offloading Uplink Traffic of Cellular Networks

**DOI:** 10.3390/s20216287

**Published:** 2020-11-04

**Authors:** Qian Wang, Juan Fang, Bei Gong, Xiaojiang Du, Mohsen Guizani

**Affiliations:** 1 College of Computer Science, Beijing University of Technology, Beijing 100124, China; wangqian2020@bjut.edu.cn (Q.W.); fangjuan@bjut.edu.cn (J.F.); 2 Department of Computer and Information Science, Temple University, Philadelphia, PA 19122, USA; dxj@ieee.org; 3 Department of Computer Science and Engineering, Qatar University, Doha 2713, Qatar; mguizani@ieee.org

**Keywords:** uplink traffic offloading, opportunistic communications, mobility prediction, mobile crowd sensing applications

## Abstract

Wi-Fi uploading is considered an effective method for offloading the traffic of cellular networks generated by the data uploading process of mobile crowd sensing applications. However, previously proposed Wi-Fi uploading schemes mainly focus on optimizing one performance objective: the offloaded cellular traffic or the reduced uploading cost. In this paper, we propose an Intelligent Data Uploading Selection Mechanism (IDUSM) to realize a trade-off between the offloaded traffic of cellular networks and participants’ uploading cost considering the differences among participants’ data plans and direct and indirect opportunistic transmissions. The mechanism first helps the source participant choose an appropriate data uploading manner based on the proposed probability prediction model, and then optimizes its performance objective for the chosen data uploading manner. In IDUSM, our proposed probability prediction model precisely predicts a participant’s mobility from spatial and temporal aspects, and we decrease data redundancy produced in the Wi-Fi offloading process to reduce waste of participants’ limited resources (e.g., storage, battery). Simulation results show that the offloading efficiency of our proposed IDUSM is $\left(56.54\times 10^{-7}\right)$, and the value is the highest among the other three Wi-Fi offloading mechanisms. Meanwhile, the offloading ratio and uploading cost of IDUSM are respectively 52.1% and $\left(6.79\times 10^{3}\right)$. Compared with other three Wi-Fi offloading mechanisms, it realized a trade-off between the offloading ratio and the uploading cost.

## 1. Introduction

With the proliferation of smart devices with various sensors (e.g., smart phones, iPads, intelligent vehicles etc.), Mobile Crowd Sensing (MCS) has become an appealing paradigm by empowering smart devices as participants to contribute data sensed or generated from them, and then aggregating and fusing these data in the cloud platform for crowd intelligence extraction and human-centric service delivery [1]. It is gradually being used in more and more large-scale sensing applications such as air condition monitoring, health monitoring, and so forth. It can also support Intelligent Transportation System (ITS) to provide innovation services, improve cost-effectiveness and efficiency of transportation and traffic management systems [2,3], for example, a bus arrival time prediction is realized relying on the participatory sensing of bus passengers [4], and a bus route optimization planning is designed based on the sensing data of participants [5].

In MCS, after participants finished assigned tasks the data should be uploaded to the backend server located in the cloud for further processing. Nowadays most existing work assumes that the data is uploaded by cellular networks as soon as the data is produced. However, according to Cisco forecast, cellular networks are very likely to become overloaded due to the dramatically increased traffic [6], even though it upgrades to the 5th Generation (5G) mobile networks with higher capacity. 5G networks are expected to handle a 1000-fold increase in capacity [7,8]. However, in the long term the emerging Internet of Things (IoT), massive Machine Type Communication (mMTC), always-connected devices such as smartphones, tablets, video-game consoles, virtual/augmented reality devices, wearable electronics introducing a huge number of connections may still cause the network to be congested [9,10,11,12].

With the increase of the number of MCS applications and participants, if all data is uploaded through cellular networks, the overload problem of cellular networks may be worsened. Therefore, there is an urgent demand to offload the traffic of cellular networks generated by the data uploading process of MCS. One promising solution for this issue is Wi-Fi uploading that source participants transmit collected data to Wi-Fi Access Points (APs) by direct or indirect short-range radios (e.g., bluetooth, Wi-Fi) with the help of encountered participants, and then the data is uploaded to the cloud through wired links of Wi-Fi APs. Through the method, part traffic of cellular networks is offloaded to Wi-Fi networks. However, the Wi-Fi uploading method might lead to uncontrollable uploading delay, since short-range radios between participants or between participants and APs are opportunistic, and the data transmission method is called opportunistic transmissions. For the data with time constraint, such as traffic condition and air quality, if the data is not uploaded to the server before it expires, the source participant will reupload the data through cellular networks to ensure the quality of the application. Hence, if the collected data fails to be uploaded through Wi-Fi uploading, not only the traffic of cellular networks will not be reduced, but also limited resources (storage, battery, etc.) of participants participating in Wi-Fi uploading process will be wasted. In addition, it may produce additional transmission cost because of participants’ data plans.

Pay As You Go (PAYG) and Unlimited Data Plan (UnDP) are two data plans widely used by most telecom operators [13,14]. A participant with a PAYG data plan means he/she needs to pay the operator according to the amount of data transferred via cellular networks. An UnDP participant can transfer unlimited amounts of data via cellular networks during a time period (usually for a month) without additional cost, because the payment for UnDP is fixed. In the Wi-Fi uploading failure scenario, if the source participant’s data plan is PAYG, he/she needs to pay additional uploading cost. However, if from the start the source participant, who cannot successfully upload the data through Wi-Fi networks, directly chooses cellular uploading, in which he/she forwards data to an UnDP participant within valid time of the data by opportunistic transmissions and the UnDP participant uploads the data by cellular networks. The additional transmission cost may be saved and part participants’ resources will not be wasted.

Therefore, in this paper we study how to design an appropriate data uploading mechanism to make a balance between the offloaded traffic of cellular networks and participants’ uploading cost considering the differences among participants’ data plans and direct and indirect opportunistic transmissions. The uploading cost contains accessing Wi-Fi APs and the transmission of cellular networks. We proposed an Intelligent Data Uploading Selection Mechanism (IDUSM). The mechanism first helps the source participant choose an appropriate data uploading manner from Wi-Fi uploading and cellular uploading based on our proposed probability prediction model, and then continues optimizing the performance objective for the chosen data uploading manner, for example, the performance objective of Wi-Fi uploading is increasing the offloaded cellular traffic and the performance objective of cellular uploading is decreasing participants’ uploading cost. The contributions of the paper can be summarized as follows:

- Our proposed IDUSM is distributed. It as a background program runs in every participant helping the participant make data uploading decision. It is feasible in large scale scenarios. Meanwhile, the proposed IDUSM realized a trade-off between the offloaded cellular networks traffic and participants’ uploading cost considering the differences among participants’ data plans and probabilities of opportunistic transmissions.
- The decision of data uploading manner in IDUSM is based on our proposed probability prediction model. The model precisely predicts a participant’s probability of successfully transferring data to a Wi-Fi AP considering direct and indirect opportunistic transmission and time variation of opportunistic contact patterns. It increases the accuracy of data uploading decision.
- The proposed IDUSM considers the waste of participants’ limited resources by the redundant data produced in the Wi-Fi uploading process. It reduces the data redundancy by only allowing participants carrying data to replicate data to participants with higher probability of contacting the Wi-Fi AP.

The rest of the paper is organized as follows—Section 2 gives a brief overview of related work. In Section 3, we describe the proposed IDUSM. Section 4 presents the participant’s mobility prediction model. Section 5 evaluates the performance of our proposed approach by simulation, and Section 6 concludes the paper.

## 2. Related Work

Nowadays, there are a lot of work studying the mobile data offloading problem from the aspect of offloading the data from the overloaded cellular networks to other networks (such as the Wi-Fi network) by opportunistic communications [15]. These work can be divided into two categories, offloading downlink traffic and uplink traffic according to the flow direction.

**Offloading downlink traffic:** according to the number of data requesters the downlink offloading works can also be divided into two categories—the data requested by many users [16,17,18,19,20,21,22,23,24,25] or by one user [26,27,28,29]. References [16,17,18,19,20,21,22,23,24,25] proposed to offload the data by selecting a subset of nodes downloaded data from cellular networks and other unselected nodes get the data by opportunistic transmissions. References [26,27,28,29] proposed methods to optimize the probability of offloading data to a specific destination.

All the methods mentioned above study how to offload downlink traffic of cellular networks. However, they are not suitable for offloading uplink traffic. Firstly, each mobile user’s data is unique in the uploading process unlike downlink offloading where many mobile users request the same data. Secondly, there is not an appointed destination for the upload offloading. All contacted Wi-Fi APs are useful for offloading uplink traffic.

**Offloading uplink traffic:** for increasing the offloaded uplink traffic of cellular networks, Reference [30] proposed a weighted proportionally fair bandwidth allocation algorithm aiming to improve the energy efficiency of the participants and increase the offloaded data volume under the concurrent use of Wi-Fi APs and cellular networks; Reference [31] proposed a spatiotemporal opportunistic transmission algorithm by analyzing the spatiotemporal visiting probabilities and similarities of nodes to achieve high offloading success rate; Reference [32] developed an energy-efficient multi-hop data forwarding method based on Dynamic Source Routing (DSR) to deliver the sensory data to participants, and then upload the data to the backend server through cellular networks. These three methods ignore the cost of data uploading.

Part of the work is proposed to reduce the cost of uploading data by opportunistic communications. The Cost-Effective Multi-Mode Offloading (CEMMO) mechanism [33] selected the most effective upload offloading mode from cellular delivery, delay tolerance delivery and peer-assisted delivery through the prediction of user mobility and connectivity with Wi-Fi APs to reduce the overall cost in terms of financial settlement, energy consumption, and user satisfaction. ecoSense [34] attempted to minimize the organizer’s data refund budget considering the two most common real-life 3G price plans UnDP and PAYG. Reference [35] proposed two algorithms including progress-balanced algorithm and social-aware forwarding algorithm to minimize total uploading cost of all users including data plan costs and extra costs for uploading data outside the plans. Reference [36] proposed the Prediction-based User Recruitment for mobile crowdsEnsing-Delegation Forwarding (PURE-DF) method by delivering data to UnDP user to minimize data uploading cost.

Some other work optimizes other metrics in addition to uploading cost. Reference [37] proposed the Cost-aware Energy efficient Offloading (CEO) policy to minimize the energy consumption within a given deadline and a monetary cost constraint. An energy-efficient and cost-effective data uploading framework effSense [13] reduced the data cost by maximally offloading data to Wi-Fi APs or encountered data-plan users. Reference [38] proposed a general Delay-Aware Wi-Fi Offloading and Network Selection (DAWN) algorithm aiming to achieve a good tradeoff between the participant’s payment and Quality of Service (QoS) characterized by the data’s valid time. Reference [39] proposed a personalized data offloading scheme to provide maximum throughput within the cellular budget through employing an adaptive model to predict the throughput of Wi-Fi APs and the network usage of participants. Reference [40] proposed a preference-oriented offloading strategy for mobile node to make a proper cost-delay tradeoff.

However, none of the above-mentioned methods simultaneously optimizes the offloaded traffic of cellular networks and participants’ cost of uploading data. In the paper, we propose the distributed IDUSM to select the appropriate data uploading manner based on the proposed probability prediction model realizing a trade-off between the offloaded traffic and the uploading cost, and meanwhile reducing unnecessary data copies in opportunistic offloading process to reduce the waste of participants’ limited resources.

## 3. The Intelligent Data Uploading Selection Mechanism

**Figure 1:**
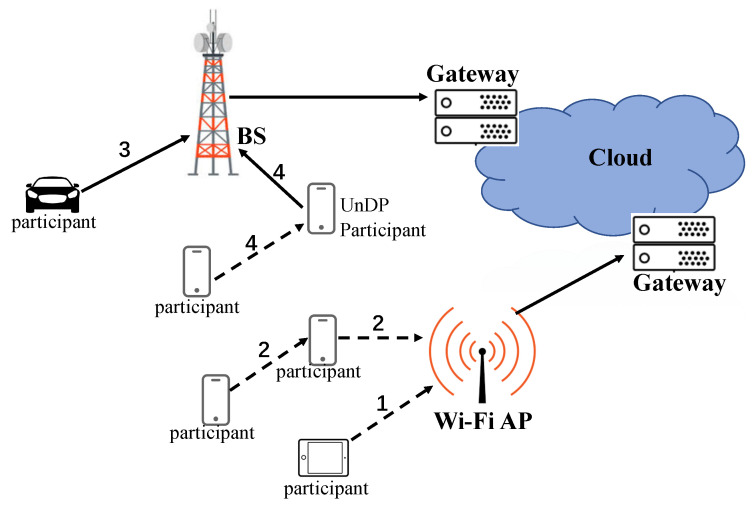
Data uploading manners.

The paper defines two data uploading manners: **Wi-Fi uploading** and **cellular uploading**, as illustrated in Figure 1, where the black dotted arrow is opportunistic transmitting and solid arrow represents non-delayed data link including cellular transmission and wired link transmission. **Wi-Fi uploading** utilizes opportunistic transmissions to offload the traffic of cellular networks to Wi-Fi networks. It contains source participants directly offload the data to Wi-Fi APs such as path 1 or indirectly offload the data to Wi-Fi APs with the help of other encountered participants such as path 2, and then the Wi-Fi networks upload the data to the cloud side by wired links. **Cellular uploading** utilizes cellular networks to upload data. It contains the source participant directly uploads data by cellular networks like path 3, or indirectly uploads data by cellular networks with the help of opportunistically encountered participants like path 4.

**Figure 2:**
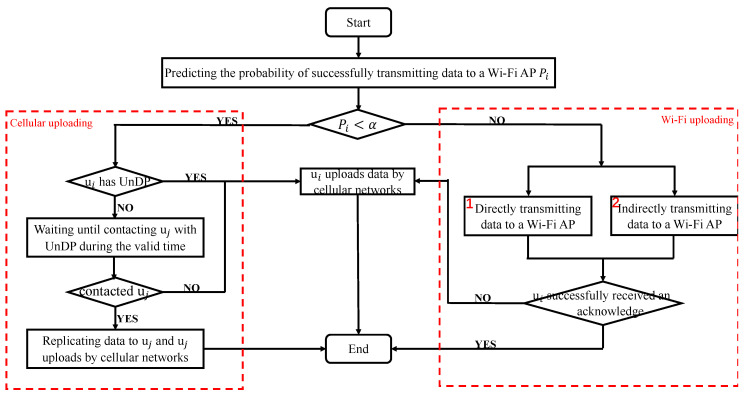
The proposed Intelligent Data Uploading Selection Mechanism (IDUSM).

Our proposed IDUSM as a background program runs in every participant. Its process is illustrated in Figure 2. It firstly helps the participant $u_{i}\left(i\in 1,2,...,n\right)$ choose an appropriate data uploading manner from Wi-Fi and cellular uploading. Wi-Fi uploading aims to offload the traffic of cellular networks, but cellular uploading aims to reduce transmission cost. For finding a balance between them, we set $u_{i}$ chooses Wi-Fi uploading when his/her probability $P_{i}$ is larger than α that means $u_{i}$ has high possibility to offload data to Wi-Fi networks, or $u_{i}$ chooses cellular uploading. $P_{i}$ represents the probability that $u_{i}$ transmits the data to a Wi-Fi AP within the valid time of the data by direct and indirect opportunistic transmissions, and it is calculated by our proposed probability prediction model (Section 4). The value of α is set by the server located in the cloud in advance. It influences the performance of proposed mechanism, and we will discuss it in Section 5.2.

If cellular uploading is chosen, $u_{i}$ will execute the following processes to reduce the cost of uploading data by cellular networks. If the data plan of $u_{i}$ is UnDP, he will directly upload the data by cellular networks, or he will wait until encountering a participant with UnDP $u_{j}\left(j\in 1,2,...,n\right)$ or at the end of the valid time of the data. If $u_{i}$ encounters $u_{j}$, he will replicate data to $u_{j}$ and $u_{j}$ uploads the data by cellular networks, or $u_{i}$ will directly upload the data by cellular networks. Through this way the transmission cost can be reduced.

If Wi-Fi uploading is chosen, $u_{i}$ will execute the following processes to increase the offloaded cellular traffic while reducing the waste of participants’ limited resources produced by unnecessary data replication. As shown in Figure 2, $u_{i}$ executes sub-process 1 and 2 in parallel, and the sub-processes end when $u_{i}$ receives an acknowledgement message or the current time exceeds the valid time of the data. If $u_{i}$ does not receive an acknowledge message within valid time of the data, he/she will directly upload the data by cellular networks. Sub-process 1 represents $u_{i}$ transmits data to directly encountered Wi-Fi APs. Sub-process 2 represents $u_{i}$ indirectly transmits the data to Wi-Fi APs with the help of other encountered participants. Since transmitting data more than 2 hops does not improve the efficiency of opportunistic transmissions, even dramatically increases data redundancy [41,42], the path of opportunistically transmitting data to a Wi-Fi AP is at most two hops.

**Figure 3:**
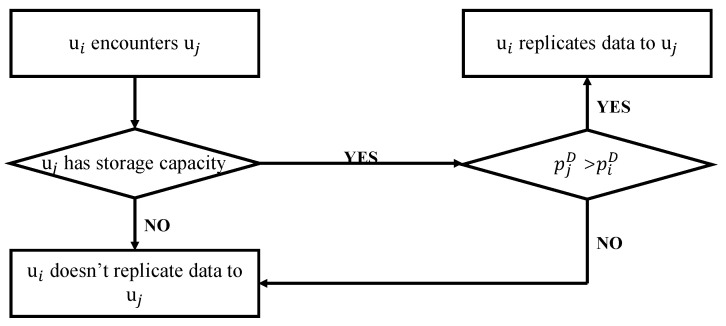
Data forwarding selection in sub-process 2.

Redundant data produced by opportunistic transmissions in sub-process 2 consumes participants’ limited resources, even data offloading ratio may be decreased. For example, there is a situation that the encountered participants do not have enough space to carry the data because of carrying other participants’ data, specifically, the encountered participant can successfully offload the data to the Wi-Fi AP but some of its carried data cannot be successfully offloaded to the Wi-Fi APs. Therefore, for reducing redundant data in sub-process 2, we propose $u_{i}$ only replicates data to the encountered participant $u_{j}$ whose probability $p_{j}^{D}$ is larger than the probability $p_{i}^{D}$. $p_{j}^{D}$ is the probability that $u_{j}$ directly transmits the data to a Wi-Fi AP within valid time of the data, and $p_{i}^{D}$ represents the probability that $u_{i}$ directly transmits the data to a Wi-Fi AP within valid time of the data calculated in Section 4.1. The detailed procedures of sub-process 2 are illustrated in Figure 3.

## 4. The Probability of Transmitting Data to a Wi-Fi AP

In the paper, we focus on offloading traffic data, which can be completely transferred within one contact, such as texts or numerical data. Hence, the probability of a participant of transmitting data to a Wi-Fi AP within the valid time of the data is same with the probability that the participant directly or indirectly contacts the Wi-Fi AP within the valid time of the data. In the section, predicting the probability contains two parts: the probability of directly contacting a Wi-Fi AP within the valid time of the data (Section 4.1) and the probability of indirectly contacting a Wi-Fi AP within the valid time of the data (Section 4.2).

**Figure 4:**
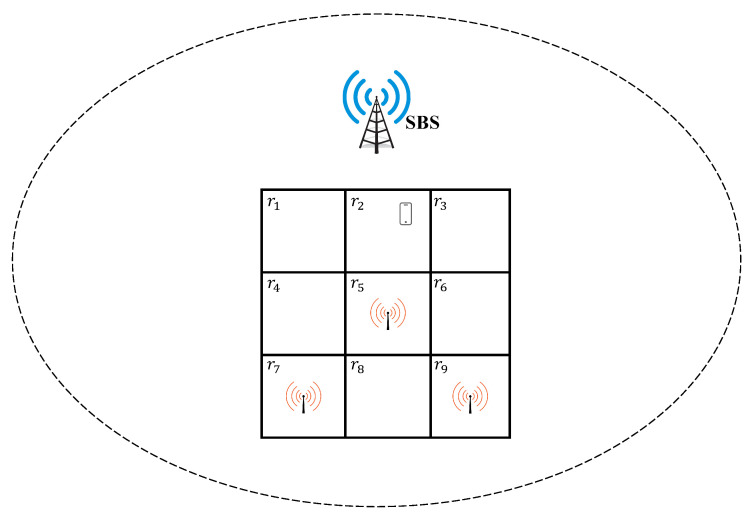
A sample scenario.

We consider a participant moves within the coverage of cellular networks such that the cellular connection is always available to the participant. Occasionally, the participant may be able to access Wi-Fi APs at some regions. This means the Wi-Fi connection is location dependent and may not be available to the participant at all time. Therefore, in the paper, we propose the Small Base Station (SBS) in cellular networks divides its covered area into small regions with the same size. The length of the diagonal of a small region should be smaller than the coverage radius of a Wi-Fi AP that ensures that a participant can connect to a Wi-Fi AP in a small region if the region has one or multiple Wi-Fi APs. A participant can get the location information of regions by its connected SBS when he/she accesses the covered area of the SBS for the first time. Figure 4 shows a participant’s sample moving scenario. The area covered by a SBS is divided into small regions with unique identifiers and a participant can contact the Wi-Fi AP only in regions $r_{5},r_{7},r_{9}$. Since References [43,44,45] observe that the movement of an individual exhibits a high degree of regularity and an individual regularly visits a small set of locations/regions and moves between those locations, in this paper we use participants’ historical contact records to predict the participants’ probabilities of directly contacting a Wi-Fi AP or indirectly contacting a Wi-Fi AP with the help of other peer participants within a specific time constraint. The specific time constraint represents the valid time of the data. A participant records his/her transition and direct contacting records based on the location information of the divided regions, and the records of indirectly contacting Wi-Fi APs with the help of contacted peer participants are got by short-range wireless communications (e.g., bluetooth) when two participants contact each other.

### 4.1. The Probability of Directly Contacting a Wi-Fi AP

Since contact patterns are time-varying, we predict the probability from spatial and temporal aspects. The spatial aspect predicts the probability that the participant moves between regions, and the temporal aspect predicts the duration of the participant of staying in a region. In this section, we integrate spatial and temporal aspects to predict the probability that a participant directly contacts a Wi-Fi AP within a specific time constraint.

#### 4.1.1. Spatial Prediction

The movement of a participant is regular. Therefore, we set the time period to express regularity, and at every time period the participant’s mobility is similar, for example, participants visit certain types of locations such as home and workplace at the same time everyday, and in this case a day is a time period. What’s more, to accurately acquire the time-dependent transition probability we split the time period into a series of small time intervals, whose durations are the same.

Based on the historical transition information, the participant uses a matrix M(t) to record his/her transition probability $p_{ij}\left(t\right)$ from a region $r_{i}\in \left\{R_{p}-R_{w}\right\}$ without a Wi-Fi AP to another region $r_{j}\in R_{p}$ during the time interval *t*, where $R_{p}\left(\right|R_{p}\left|=m\right)$ represents regions that are visited at least *x* time periods aiming to eliminate regions visited randomly, and $R_{w}\left(\right|R_{w}\left|=m^{{}^{\prime }}\right)$ is the Wi-Fi available regions. The parameter *x* is set by the participant based on real data sets in advance. The transition probability $p_{ij}\left(t\right)$ is calculated as follows:

(1) $$p_{ij}\left(t\right)=N\left(r_{i}r_{j},t\right)/N\left(r_{i},t\right),$$

where $N(r_{i}r_{j},t)$ and $N(r_{i},t)$ are statistics based on historical records of the participant; $N(r_{i},t)$ is the number of the participant visiting region $r_{i}$ during *t*; $N(r_{i}r_{j},t)$ is the number of the participant moving from $r_{i}$ to $r_{j}$ during *t*; when $r_{j}=r_{i}$, $N(r_{i}r_{j},t)$ represents the number of the participant staying in $r_{i}$ during *t*. Since a participant only stays in its original region or moves to adjacent regions during a time interval, for example, the participant can only stays in $r_{2}$ or move to its adjacent regions $r_{1},r_{3},r_{5}$ at the current time interval in Figure 4, the probabilities of moving to non-adjacent regions are 0. The probability calculation method refers to Reference [33].

#### 4.1.2. Temporal Prediction

Temporal prediction gets the duration that a participant stays in a region before making a transition to some other regions at a time interval. Using historical records of previous durations, we estimate probabilities of the durations in a region at a time interval. We act the duration, which has maximum probability, as the participant’s staying time in the region at the time interval. The detailed calculation method is described as follows:

$H_{i}\left(t\right)=\left\{d|d\in \left\{1,2,3,...\right\}\right\}$ represents historical records of previous duration of the participant in the region $r_{i}$ at *t*, where *d* is the number of time intervals which is divided by duration. $p_{i}^{t}\left(d\right)$ calculated by (2) represents the probability that at *t* the participant stays in the region $r_{i}$*d* time intervals, where $N(r_{i}t,d)$ is the number of the participant staying in $r_{i}$ for *d* time intervals at *t*. We choose *d* whose probability is the largest from the set $\left\{p_{i}^{t}\left(d\right)|d\in H_{i}\left(t\right)\right\}$ as the staying duration of $u_{i}$ in region $r_{i}$ at *t*.

(2) $$p_{i}^{t}\left(d\right)=N\left(r_{i}t,d\right)/\sum_{d\in H_{i}\left(t\right)}N\left(r_{i}t,d\right).$$

#### 4.1.3. The Combination of Spatial and Temporal Prediction

A participant may need to move to other regions to contact a Wi-Fi AP, for example in Figure 4, although the participant cannot contact a Wi-Fi AP at the current time interval, he can contact a Wi-Fi AP through multiple movements such as $r_{2}\to r_{5}$ or $r_{2}\to r_{1}\to r_{4}\to r_{5}$ or $r_{2}\to r_{1}\to r_{4}\to r_{7}$, and so forth. Thus, a participant’s probability of contacting a Wi-Fi AP within a specific time constraint should be the sum of probabilities of multiple movements.

The movement of a participant in a set of possible regions following a Markovian mobility model, which means the next visiting region of a participant only depends on the previous region. The model is widely used in the literature [33,38,46], hence we also use a first-order Markov model as the spatial prediction model to predict the probability of the participant of moving to the next region. Moreover, since the probability of moving from a region to another depends on time, our proposed probability prediction model combines spatial and temporal prediction. We describe one step transition probability of a participant in the following.

We use $r_{s}$ to record the starting region of the participant; *t* to record the current time instance located time interval; $t_{e}$ to record the time interval that the deadline of the time constraint located in. We get the number of the participant’s staying time intervals *d* based on the temporal prediction method (Section 4.1.2). Thus, the time interval that the participant moves to the next region is (t+d) and the transition probability matrix is M(t+d). If $\left(\left(t+d\right)<t_{e}\right)$, the participant will move to the next possible adjacent region $r_{a}$, and the probability $p_{a}\left(t+d\right)$ that the participant reaches the region $r_{a}$ is calculated by (3),

(3) $$p_{a}\left(t+d\right)=p_{s}\left(t\right)\cdot p_{s}^{t}\left(d\right)\cdot p_{sa}\left(t+d\right),$$

where $p_{s}\left(t\right)$ is the probability that the participant reaches the region $r_{s}$ at the time interval *t*; $p_{s}^{t}\left(d\right)$ represents the probability of staying in the region $r_{s}$ for *d* time intervals; $p_{sa}\left(t+d\right)$ gotten from M(t+d) is the probability that the participant moves from $r_{s}$ to $r_{a}$ at the time interval (t+d).

**Figure 5:**
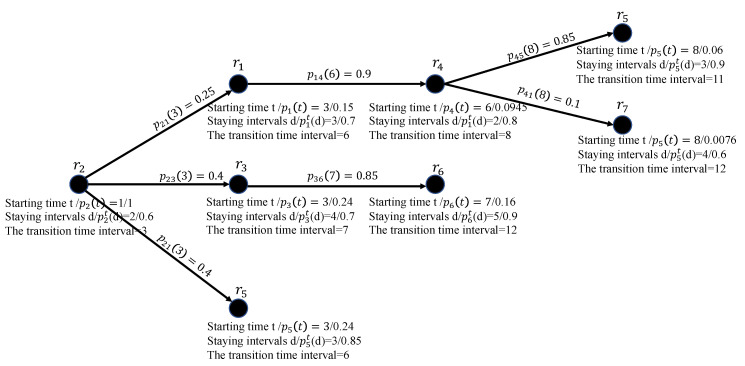
An example of Algorithm 1.

Since a participant’s next region has some possibilities, he/she might contact a Wi-Fi AP by multiple movements, for example, in Figure 4 the participant can contact a Wi-Fi AP by directly moving to the region $r_{5}$ or multiple movements like $r_{1}\to r_{4}\to r_{7}$ or $r_{3}\to r_{6}\to r_{9}$. For calculating the probability that a participant directly contacts a Wi-Fi AP within a time constraint, we need to traverse all possible movements during the time constraint. Hence we recursively call our proposed one step transition probability calculation method, where every possible next region, which do not contain a Wi-Fi AP as the starting region, to calculate the participant’s probability of directly contacting the Wi-Fi AP within a time constraint. An iteration ends when the time interval exceeds the time interval that the deadline of the time constraint located in or the participant reaches a region contained a Wi-Fi AP, and the probability will be added to $p_{i}^{d}$ if the participant arrives the region with a Wi-Fi AP within the specific time constraint. The above-mentioned procedures end when all possible next regions are traversed, and we get $p_{i}^{d}$ ultimately. The whole process is described in Algorithm 1.
**Algorithm 1** Predicting the probability of $u_{i}$ directly contacting a Wi-Fi Access Point (AP) within a time constraint**Input:** the starting region $r_{s}$, the starting time interval *t*, the stop time interval $t_{e}$. **Output: **
$P_{i}^{D}$ **Initialize: **
$P_{i}^{D}=0,p_{s}\left(t\right)=1$ 1: **OneStepTransition()** { 2: **if** ($r_{s}$ contains a Wi-Fi AP) **then** 3: $\;P_{i}^{D}+=p_{s}\left(t\right)$; 4: **else** 5:  Getting the staying time intervals *d* and $p_{s}^{t}\left(d\right)$ using (2); 6:  t+=d; 7:  **if**
$\left(t<t_{e}\right)$
**then** 8:   **for** every possible next region $r_{a}$
**do** 9:    Calculating $p_{sa}\left(t\right)$ using (1); 10:   Calculating $p_{a}\left(t\right)$ using (3); 11:   $r_{s}=r_{a}$; 12:   $p_{s}\left(t\right)=p_{a}\left(t\right)$; 13:   Calling **OneStepTransition()** 14:  **end for** 15: **end if** 16: **end if** } 17: **return**
$P_{i}^{D}$;


We use an example to illustrate the Algorithm 1. The moving scenario of the participant $u_{1}$ is shown in Figure 4. His start region is $r_{2}$. The current time interval is 1 and the stop time interval is 9. The whole processes of the Algorithm 1 is shown in Figure 5. Since $p_{2}^{1}\left(2\right)$ is the largest among other possible staying durations, $u_{1}$ stays in $r_{2}$ for 2 time intervals, and then $u_{1}$ moves to the next adjacent region at the 3rd time interval. $u_{1}$ finds he has three choices: $r_{1},r_{3},r_{5}$, whose probabilities are not zero based on his transition matrix. We found the summary of probabilities of arriving all possible regions ($r_{1},r_{3},r_{5}$) is not 1, because the participant also randomly moves to other regions besides the periodic visiting regions. If $u_{1}$ reaches $r_{1}$, he will continue moving until reaching $r_{5}$ or $r_{7}$, because $r_{1}$ and $r_{4}$ do not contain a Wi-Fi AP and the transition time interval is smaller than the stop time interval. The probabilities of reaching $r_{5}$ and $r_{7}$ are added to $P_{1}^{D}$, and this branch ends. If $u_{1}$ arrives $r_{3}$, he will move to the next region since $r_{3}$ does not contain a Wi-Fi AP and the transition time interval is smaller than the stop time interval. Based on the transition matrix $u_{1}$’s next step has only one choice: $r_{6}$. However, when $u_{1}$ reaches $r_{6}$, the branch will end since the transition time interval exceeds the stop time interval. What’s more, since $r_{5}$ contains a Wi-Fi AP, $u_{i}$’s probability of arriving $r_{5}$ is directly added to $P_{1}^{D}$ and this branch ends. Until now, all possible arriving regions are traversed and the whole process ends. $P_{1}^{D}$ is equal to 0.31.

### 4.2. The Probability of Indirectly Contacting a Wi-Fi AP

**Figure 6:**
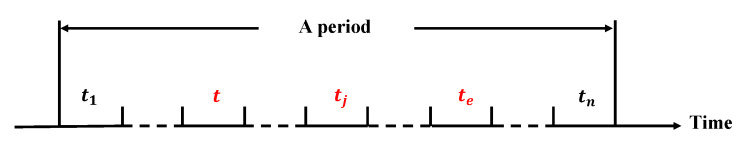
The position of $t_{j}$.

Calculating the probability $p_{i}^{I}$ that a participant $u_{i}\left(i\in \left\{1,2,...,n\right\}\right)$ indirectly contacts a Wi-Fi AP with the help of other contacted peer participants within a specific time constraint includes two parts. The first is calculating the probability of contacting a peer participant $u_{j}$ at the time interval $t_{j}$, which is smaller than the stop time interval $t_{e}$, as illustrated in Figure 6 where *t* is the starting time interval. Since transmitting data more than 2 hops does not improve the forwarding efficiency, even dramatically increases data redundancy, the second part is to calculate $u_{j}$’s probability of directly contacting a Wi-Fi AP and the starting time interval is $t_{j}$.

$u_{i}$ calculates the probability $p_{i}^{I}$ based on his historical contact records $<u_{j},n_{j},t_{j},r_{j}>$, which represents the participant contacts $u_{j}$$n_{j}$ times at the time interval $t_{j}$ in the region $r_{j}$. For every historical contact record, the chosen contacted participant $u_{j}$ should satisfy $\left(n_{j}>x\right)$ to eliminate the influence of randomly contacted participants and $\left(t_{j}<t_{e}\right)$ to limit the contacted time. The parameter *x* is defined in Section 4.1.1. The probability $p_{j}^{j}\left(t_{j}\right)$ that $u_{i}$ encounters $u_{j}$ at the time interval $t_{j}$ in the region $r_{j}$ is calculated by (4), where *n* is the sum of all contact times in historical contact records. After calculating the probability $p_{j}^{j}\left(t_{j}\right)$, we need to calculate the probability $p_{j}^{D}$ that $u_{j}$ directly contacts a Wi-Fi AP within the rest of time. $p_{j}^{D}$ is calculated through calling the Algorithm 1, and the input parameters including the starting region, the starting time interval and the stop time interval are $r_{j}$, $t_{j}$ and $t_{e}$ respectively. $p_{i}^{I}$ is calculated using (5). The whole process is described in Algorithm 2.

(4) $$p_{j}^{j}\left(t_{j}\right)=n_{j}/n;$$

(5) $$p_{i}^{I}=p_{i}^{I}+p_{j}^{j}\left(t_{j}\right)\cdot p_{j}^{D}.$$

We integrate the above-mentioned two methods of calculating the probability of directly contacting a Wi-Fi AP $p_{i}^{D}$ and the probability of indirectly contacting a Wi-Fi AP $p_{i}^{I}$ to get the probability $P_{i}$ that the participant $u_{i}$ contacts a Wi-Fi AP within a specific time constraint shown in (6). In conclusion, the participant executes our proposed probability calculation algorithm when he/she has data to be uploaded, and the computation complexity of the algorithm is $O(\frac{T}{D_{s}}\cdot \left(N_{e}+1\right))$, which is decided by the number of time intervals *T* split by the valid duration of the data, the number of encountered participants $N_{e}$ during the valid time of the data, and the average number of time intervals $D_{s}$ staying in a region. Moreover, the probability is calculated based on the participants’ historical contact records. The participant directly records contacted Wi-Fi APs and peer participants, and the records of indirectly contacting Wi-Fi APs are got by short-range wireless communications when the participant contacts peer participants. The methods of getting information do not occupy communication resources of cellular networks.

(6) $$P_{i}=p_{i}^{D}+p_{i}^{I}.$$

**Algorithm 2** Predicting the probability of $u_{i}$ indirectly contacting a Wi-Fi AP within a time constraint**Input:** the starting region $r_{s}$, the starting time interval *t*, the stop time interval $t_{e}$. **Output: **
$p_{i}^{I}$ **Initialize: **
$p_{i}^{I}=0,p_{s}\left(t\right)=1$ 1: **for** every historical records of $u_{i}$
**do** 2:  **if** ($\left(t<t_{j}<t_{e}\right)$&$\left(n_{j}>x\right)$) **then** 3:   Calculating $p_{j}^{j}\left(t_{j}\right)$ using (4); 4:   Calling Algorithm 1($r_{j},t_{j},t_{e}$) to get $p_{j}^{D}$; 5:   $p_{i}^{I}+=p_{j}^{j}\left(t_{j}\right)\cdot p_{j}^{D}$; 6:  **end if** 7: **end for** 8: **return**
$p_{i}^{I}$;


## 5. Performance Evaluation

In this section, we first describe the setup of simulation environment and performance evaluation metrics, then discuss the value of α in our proposed IDUSM, and finally we compare performance of our proposed IDUSM with three related offloading mechanisms under different simulation scenarios.

### 5.1. Environment Setup

The simulation is executed on the ONE simulation platform [47]. In the simulation, there are 200 participants with UnDP and PAYG data plan, and the exact number of each kind is varied based on different experiments. The participants move within a 4.5 km × 3.5 km section of Helsinki, Finland, and they follow the Working Day Movement model, which is a realistic mobility model that simulates the daily mobility of people. A cellular BS locates at the center of the map and covers all the users within the field. Wi-Fi APs are randomly located with the sites in the map, and the number of Wi-Fi APs is varied based on different experiments.

Every participant executes a Poisson process to fire an event for data generation, where the interval between two sequential events follows the Poisson distribution with parameter λ = 1800 s. The size of data is 2 MB. We also set the bandwidth of BS and Wi-Fi AP are unlimited that ensure the data can be completely transferred during a contact. Considering participants’ storage capacities, we set their caches are 50 MB. For evaluating the cost of transferring data, we assume the usage price of cellular networks of the participant with PAYG data plan is 1 RMB/MB and the participant with UnDP is free. The cost of transferring data through the Wi-Fi AP is 0.05 RMB/MB. The values of uploading cost of PAYG participant and Wi-Fi AP aims to express their ratio referred to References [33,48]. They are not the real cost.

In all experiments, we set the region size is equal to 300 m × 340 m, and time interval duration is equal to 10 min. It corresponds to 150 regions and 144 distinct time intervals. The total duration of our experiment is 20 days. We use 10 days to train, which aims to build the prediction model, and the rest 10 days to evaluate. We evaluate the performance from the following three metrics:

1. Offloading ratio: is used to measure the offloaded cellular network traffic. It is expressed by the fraction of the total amount of generated data that is offloaded through Wi-Fi networks shown as follow:

offloading ratio = (Total data offloaded through Wi-Fi networks)/(Total data generated)

2. Offloading efficiency: Offloading ratio is improved by increasing data redundancy in the network, but the transmission of redundant data wastes participants’ limited resources such as storage and battery. In the paper, each generated data or replicated data is referred to as a data segment. We use offloading efficiency, which is represented by the fraction of offloading ratio to the number of data segments in the network, to measure the efficiency of an offloading mechanism shown as follow:

offloading efficiency = (Offloading ratio)/ (The number of data segments in the network)

3. Uploading cost: is the summary of the data uploading cost of all participants. A participant’s uploading cost contains the cost of using cellular networks and Wi-Fi APs to upload the data shown as follows:

$$\text{uploading}\;\text{cost}=\sum_{n}(C_{c}\cdot D+C_{w}\cdot D^{{}^{\prime }}),$$

where *n* is the number of participants; $C_{c}$ and $C_{w}$ represent transmission cost of cellular networks and the Wi-Fi APs respectively; *D* and $D^{\prime }$ is the size of data uploaded through cellular networks and Wi-Fi APs.

### 5.2. Performance of the Proposed IDUSM

**Figure 7:**
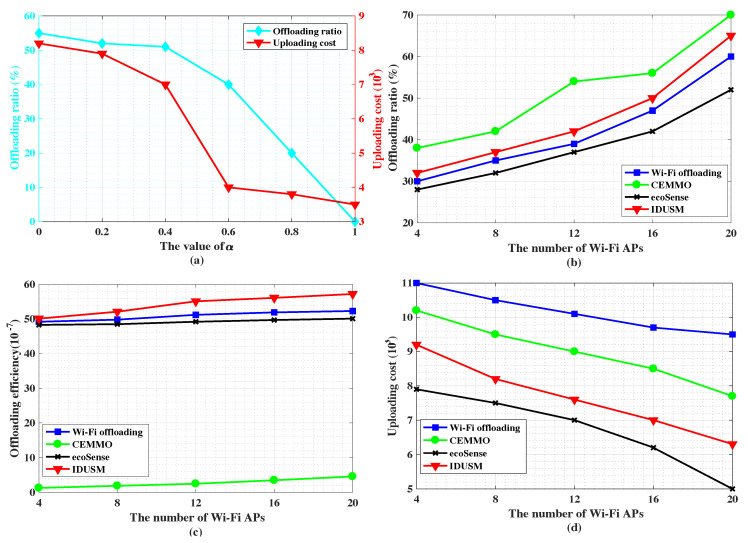
(**a**) The influence of the value of α on the performance of IDUSM. (**b**) The impact of the number of Wi-Fi APs on offloading ratio. (**c**) The impact of the number of Wi-Fi APs on offloading efficiency. (**d**) The impact of the number of Wi-Fi APs on uploading cost.

For our proposed IDUSM, we discuss the value of α, which is used to decide the selection of Wi-Fi or cellular uploading manner realizing a trade-off between the offloading ratio and the uploading cost. We set the valid time of the data is 2 h. The number of participants with UnDP and PAYG is 100 respectively. The number of Wi-Fi APs is 20. As shown in Figure 7a, we find that when (α<0.6), with the increase of α the offloading ratio decreases slowly, but the uploading cost decreases rapidly. The reason is the increase of α makes some participants, who cannot successfully upload data through Wi-Fi uploading, directly choose cellular uploading, which opportunistically forwards data to contacted UnDP participants and utilizes the UnDP participants upload the data by cellular networks. Through this way the uploading cost produced by the failure of Wi-Fi uploading is reduced, but its influence on the offloading ratio is slight.

When (α≥0.6), with the increase of α, more and more participants, who can upload data through Wi-Fi uploading, are forced to choose cellular uploading. However, some of the participants with PAYG data plan cannot contact UnDP participants during the valid time of the data. They have to upload the data using their network traffic, which leads to a slow decline in uploading cost. Therefore, when (α≥0.6), the offloading ratio sharply decrease and the uploading cost decreases slowly with the increase of α.

In conclusion, the value of α influences the performance of the proposed IDUSM, and its value should be set based on different experiment scenarios. We set (α=0.6) to achieve the optimal performance in this experiment environment.

### 5.3. Performance Comparison with Other Mechanisms

In this paper, we compare the performance of our proposed IDUSM with three related offloading mechanisms: Wi-Fi Offloading [49], CEMMO [33] and ecoSense [34] from the aspects of offloading ratio, offloading efficiency and uploading cost.

Wi-Fi Offloading: in addition to directly transfer data, participants also indirectly transfer data to Wi-Fi APs with the help of contacted peer participants. The data can be transferred to Wi-Fi APs through multi-hops. If the data is not transferred to the destination within its valid time, it will be uploaded through cellular networks.

CEMMO: is a centralized mechanism and does not distinguish participants’ uploading cost. It contains three modes of uploading: cellular delivery, direct Wi-Fi offloading and peer-assisted Wi-Fi offloading. Direct Wi-Fi offloading means the participant carries the data without forwarding until contacting a Wi-Fi AP. In peer-assisted Wi-Fi offloading mode, the participant will flood data to participants in a passing region where the summary of participants’ probabilities is the maximum among other passing regions, and the participants offload data to Wi-Fi APs. If the cost of direct offloading is smaller than peer-assisted offloading, direct offloading will be chosen, or peer-assisted offloading mode will be chosen. If the data does not be uploaded to the cloud within its valid time through direct and peer-assisted offloading, it will be uploaded through cellular delivery.

ecoSense: aims to minimize the uploading cost. If a participant has UnDP, he/she directly uploads data by cellular networks, or he/she uploads data by the OneHopFlooding strategy that the PAYG participant would relay data to another directly contacted PAYG participant until he/she meets either one of the two following stopping criteria: (1) he/she directly encountered an UnDP participant or (2) the server notifies that he/she could stop flooding.

The performance of mechanisms is evaluated under different simulation environment. Parameters used to control simulation environment are the density of Wi-Fi APs, the valid time of data, the ratio of UnDP participants’ numbers to PAYG participants’ numbers represented by β.

**Figure 8:**
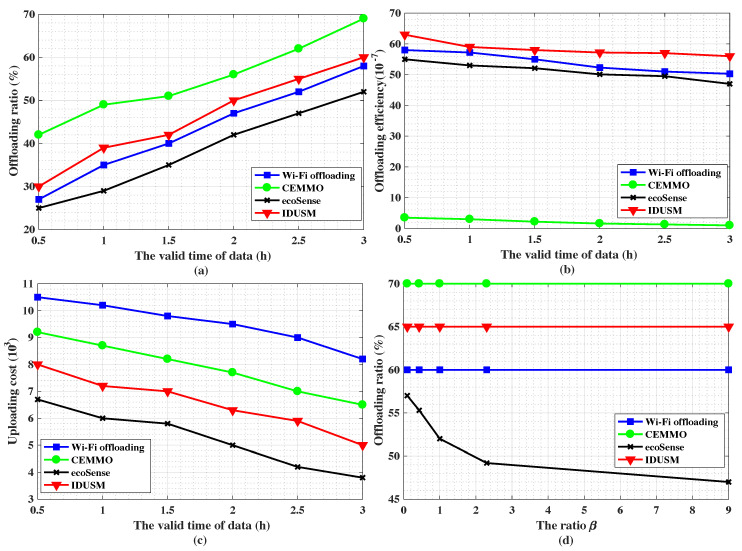
(**a**) The impact of the valid time of data on offloading ratio. (**b**) The impact of the valid time of data on offloading efficiency. (**c**) The impact of the valid time of data on uploading cost. (**d**) The impact of the ratio β on offloading ratio.

**Figure 9:**
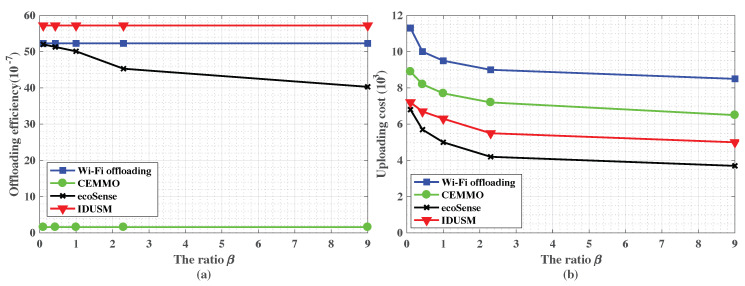
(**a**) The impact of the ratio β on offloading efficiency. (**b**) The impact of the ratio β on uploading cost.

As shown in Figure 7b,c, Figure 8a,b,d and Figure 9a, firstly, the offloading ratio of CEMMO outperforms other three Wi-Fi offloading mechanisms no matter how parameters change and its average value is 58.9%, since it makes source participants indirectly transmit data to Wi-Fi APs by flooding the data to participants in a region besides directly transmitting data, but other three Wi-Fi offloading mechanisms only replicates data to encountered parts of participants, therefore, the offloading efficiency of CEMMO is the lowest among all offloading mechanisms, and its average value is $\left(1.75\times 10^{-7}\right)$. Secondly, the average values of offloading ratio and offload efficiency of our proposed IDUSM are respectively 3.4% and $\left(4.9\times 10^{-7}\right)$ larger than Wi-Fi Offloading mechanism in all simulation scenarios, since in the process of opportunistic offloading besides accurate mobility prediction our proposed IDUSM only replicates data to encountered participants whose probabilities are higher than carried data participants, that reduces the situation that encountered participants do not have enough space to carry the data because of carrying other undelivered data. Thirdly, the average values of offloading ratio and offloading efficiency of Wi-Fi Offloading are respectively 5% and $\left(2.99\times 10^{-7}\right)$ larger than ecoSense mechanism, since ecoSense aims to reduce uploading cost but Wi-Fi Offloading focuses on traffic offloading. It is worth noting that although the values of offloading efficiency of mechanisms are almost same, the tiny gap reflects the huge gap in the number of redundant data generated by mechanisms.

The total uploading cost is related with offloading ratio and uploading participants’ data plans. As shown in Figure 7d, Figure 8c and Figure 9b, the uploading cost sorting from cheap to expensive is ecoSense, IDUSM, CEMMO and Wi-Fi Offloading mechanism in all simulation scenarios, and the average values are respectively $\left(5.68\times 10^{3}\right)$, $\left(6.79\times 10^{3}\right)$, $\left(8.18\times 10^{3}\right)$, $\left(9.78\times 10^{3}\right)$. The reasons are illustrated as follows. Firstly, in ecoSens mechanism participants with UnDP directly upload data by cellular networks aiming to minimize uploading without considering offloading the traffic of cellular network, but in our proposed IDUSM, participants with high probability of offloading data to the Wi-Fi AP will choose Wi-Fi offloading. Not only the cellular traffic is offloaded, but also the uploading cost is reduced because the uploading cost of Wi-Fi APs is low in IDUSM. However, the cost of uploading data by participants with UnDP is free, therefore, uploading cost of ecoSense is lower than IDUSM. Secondly, the uploading cost of the proposed IDUSM is lower than CEMMO mechanism, since CEMMO does not consider differences among participants’ data plans. Finally, the uploading cost of CEMMO is lower than Wi-Fi offloading mechanism, since the offloading ratio of CEMMO is bigger than Wi-Fi offloading mechanism and the transmission cost of Wi-Fi APs is much cheaper than cellular networks.

In the following, we illustrate changes of performance of mechanisms and reasons of the changes under different simulation environment in detail.

#### 5.3.1. Impact of the Density of Wi-Fi APs

In the experiment, we evaluate the impact of the density of Wi-Fi APs on the performance of Wi-Fi Offloading, CEMMO, ecoSense and our proposed IDUSM. We set the valid time of data is 2 h. The number of UnDP and PAYG participants is 100, respectively. The number of Wi-Fi APs ranges from 4 to 20. According to Figure 7b, the offloading ratios of all mechanisms increase with the increase of the number of Wi-Fi APs, since participants’ possibilities of offloading data to Wi-Fi APs are increased. At the same time, the total cost decreases with the increase of the number of Wi-Fi APs shown in Figure 7d, because more data is uploaded through Wi-Fi APs, whose uploading cost is cheap.

For Wi-Fi Offloading, ecoSense, IDUSM and CEMMO, the increase of the density of Wi-Fi APs makes participants more quickly encounter Wi-Fi APs so that the duration of replicating data to encountered peer participants becomes short, therefore the number of data in the network decreases as the increase of Wi-Fi APs. Therefore, with the increase of the density of Wi-Fi APs, the offloading efficiency of Wi-Fi Offloading, CEMMO, ecoSense and IDUSM increase illustrated in Figure 7c.

#### 5.3.2. Impact of the Valid Time of Data

The valid time of data plays an importance role in the process of data offloading. In the experiment, we set the number of Wi-Fi APs is 20. The number of UnDP user and PAYG user is equal to 100 respectively. Data’s valid time ranges from 0.5 to 3 h. Figure 8a shows that the offloading ratio of all mechanisms increases with the increase of the delay tolerant time of the data, since participants’ probabilities of encountering Wi-Fi APs increase, and meanwhile, the uploading cost of all mechanisms decreases because the transmission cost of Wi-Fi APs is cheap shown in Figure 8c.

What is more, Figure 8b shows the change of offloading efficiency with the increase of the valid time of data. For all offloading mechanisms, the number of data in the network increases as the increase of the data’s valid time, because the duration of replicating data to participants increases and the participants carried data encounters more peer participants in this process. The increased number of data in the network is much larger than the increased offloading ratio, thus the offloading efficiency of Wi-Fi Offloading, ecoSense, CEMMO and IDUSM decreases with the increase of the valid time of data.

#### 5.3.3. Impact of the Ratio β

In this experiment, we examine the influence of the ratio of UnDP participants’ number to PAYG participants’ number on the performance of all mechanisms. We set the number of Wi-Fi APs is 20. Data’s valid time is 2 h. The number of all participants is 200, and the number of UnDP participants varies from 20 to 180.

In Figure 8d and Figure 9a, for Wi-Fi Offloading and our proposed IDUSM, the selection of data uploading method and replicating data to the encountered participant only consider the probability of encountering Wi-Fi APs. The cost of transferring data would not influence these processes. For CEMMO, although the source participant selects the data uploading method based on transmission cost, it acts all participants’ transmission cost through cellular networks as the same without considering their differences. Hence, for Wi-Fi Offloading, CEMMO and our proposed IDUSM, the offloading ratio and offloading efficiency will not change with the increase of β. For ecoSense, with the increase of β, more and more participants with UnDP upload data by cellular uploading instead of Wi-Fi offloading, therefore, the offloading ratio decreases. However, in the situation the number of data in the network does not change, therefore, the offloading efficiency also decreases.

As shown in Figure 9b, all mechanisms’ actual uploading cost decreases with the increase of β, since data has greater probability of uploading through UnDP participants beside Wi-Fi uploading, that does not need to pay for the use of cellular networks.

In conclusion, the offloading efficiency of our proposed IDUSM is the highest among all offloading mechanisms, and its average value is $\left(56.54\times 10^{-7}\right)$. The offloading ratio and uploading cost of IDUSM are not optimal in all experiments. The average value of offloading ratio of CEMMO is 6.53% larger than IDUSM, and the average uploading cost of ecoSense is $\left(1.11\times 10^{3}\right)$ higher than IDUSM. However, at the same time, the average uploading cost of CEMMO is also $\left(1.39\times 10^{3}\right)$ higher than IDUSM and the average value of offloading ratio of ecoSense is 9.27% lower than IDUSM. Therefore, the experimental results prove our proposed IDUSM realized a trade-off between the offloading ratio and the uploading cost.

## 6. Conclusions and Future Work

In this paper, we studied how to design an appropriate data uploading mechanism to offload cellular traffic produced by the uploading process of mobile crowd sensing applications, and the proposed the IDUSM executed in every participant’s background to make a balance between the offloaded cellular traffic and the uploading cost. Simulation results proved that the offloading efficiency of our proposed IDUSM outperforms other three offloading mechanisms. Meanwhile, although the offloading ratio and uploading cost of IDUSM are not optimal in all experiments, it realized a trade-off between the offloading ratio and the uploading cost. However, our proposed IDUSM only considers that the data can be completely transferred during one contact. It is not suitable for scenarios in which participants need to upload the data with large size, such as video. Therefore, in the future we will consider offloading data with large size, and focus on maximizing the probability of offloading data to Wi-Fi APs by fragmenting the data and assigning the fragments to different direct and indirect paths generated by opportunistic contacts.

There are still many interesting open research areas about traffic offloading needed to be investigated. One focus is to offload downlink traffic requested by many users. Some interesting approaches to solve the issue are user selection [21,50,51] and edge caching [52,53,54,55]. User selection means the selected users download the data by cellular networks, and other requested users get the data from the selected users by short-range wireless communications. Edge caching offloads the downlink traffic by caching popular contents on edge nodes, and the requested users get the data by wireless communications. In addition, with the popularity of Artificial Intelligence (AI), utilizing AI technology to solve the problem of traffic offloading is also a meaningful topic to research [56,57].
